# Methodological approaches to help unravel the intracellular metabolome of *Bacillus subtilis*

**DOI:** 10.1186/1475-2859-12-69

**Published:** 2013-07-11

**Authors:** Hanna Meyer, Hendrikje Weidmann, Michael Lalk

**Affiliations:** 1 Institute of Biochemistry, Ernst-Moritz-Arndt-University Greifswald, Felix-Hausdorff-Strasse 4, 17487 Greifswald, Germany; 2 Interfaculty Institute for Genetics and Functional Genomics, Department of Functional Genomics, University Medicine Greifswald, Ernst-Moritz-Arndt-University Greifswald, Friedrich-Ludwig-Jahn-Str. 15a, 17489 Greifswald, Germany; 3 Current address: Oncotest GmbH, Am Flughafen 12-14, Freiburg, 79108 Baden-Württemberg, Germany

**Keywords:** *Bacillus subtilis*, Metabolomics, Sampling, Energy charge, Quenching, Filtration

## Abstract

**Background:**

*Bacillus subtilis* (*B*. *subtilis*) has become widely accepted as a model organism for studies on Gram-positive bacteria. A deeper insight into the physiology of this prokaryote requires advanced studies of its metabolism. To provide a reliable basis for metabolome investigations, a validated experimental protocol is needed since the quality of the analytical sample and the final data are strongly affected by the sampling steps. To ensure that the sample analyzed precisely reflects the biological condition of interest, outside biases have to be avoided during sample preparation.

**Results:**

Procedures for sampling, quenching, extraction of metabolites, cell disruption, as well as metabolite leakage were tested and optimized for *B*. *subtilis*. In particular the energy status of the bacterial cell, characterized by the adenylate energy charge, was used to evaluate sampling accuracy. Moreover, the results of the present study demonstrate that the cultivation medium can affect the efficiency of the developed sampling procedure.

**Conclusion:**

The final workflow presented here allows for the reproducible and reliable generation of physiological data. The method with the highest qualitative and quantitative metabolite yield was chosen, and when used together with complementary bioanalytical methods (i.e., GC-MS, LC-MS and ^1^H-NMR) provides a solid basis to gather information on the metabolome of *B*. *subtilis*.

## Background

As an essential component of the functional genomics approach, metabolomics focuses on the quantitative and qualitative analysis of small molecules [1,2]. Of central importance in metabolomics is the development of detailed protocols for reproducible sample preparation, thus allowing for the generation of useful, physiological data [3-6]. More recently, the optimization of the analytical platform and final data analysis have been a major focus. In the past years numerous improvements for ^13^C- and ^1^H-NMR, GC-MS, GCxGC-MS, LC-MS, LC-MS/MS, as well as CE/MS, have been reported. In addition, new convenient tools for data analysis and visualization have been developed, such as mzMINE [7,8] and XCMS for MS data [9]; GAVIN [10] and Metab [11] for GC-MS analysis; BATMAN [12] and BQuant [13] for NMR analysis as well as, MetAssimulo [14] and MBRole [15], MetExplore [16] and MSEA [17] to handle and visualize data sets of increasing size.

Sampling and pre-analysis represent the first steps in the generation of metabolome data, so validated sampling protocols are key to obtain reliable data. In order to ensure that samples truly reflect the physiological situation at the moment of sampling, the introduction of metabolic changes during pre-analysis must be avoided. Taking into account the rapid turnover of certain metabolites such as ATP, rapid sampling and quenching procedures are essential. Commonly used sampling techniques for metabolomics are: I) direct quenching [18], II) centrifugation in the cold [19] and III) fast vacuum dependent filtration [20], whereby the latter method appears to be the most useful. For the first method (I), leakage of intracellular metabolites due to cell lysis during organic solvent treatment is the major drawback [5,21,22]. Indeed, as several bacteria are disrupted by organic solvents, the direct quenching method is not applicable in these cases, and this method was not used in the present study. A considerable disadvantage of the method involving centrifugation in the cold (II) is a substantial time factor, and inducing physiological stress during centrifugation [23]. This method was used as “control method” in the present study. The fast filtration method (III) overcomes both drawbacks. It is fast and, as cells have already been separated from the medium during treatment with organic solvent, there is no leakage problem.

An established parameter to evaluate the sampling quality is the adenylate energy charge (EC) [23-28], which represents an index of the energy state of a cell and is defined as (ATP+½ADP)/ (AMP+ADP+ATP). A drop in the ATP concentration and/or an increase of the AMP concentration causes a decreased EC. Since most adaptation processes are ATP depended, stress induction accompanies a decrease of ATP and therewith the EC. For growing and non-stressed cells, the EC is in the range of 0.8-0.95 [24]. In metabolomics, the EC is used to monitor the cellular stress status, where stress induction during sampling is reflected in metabolic changes. Despite the fact that the EC represent one of the most important criteria for evaluating sampling methodologies, a number of published procedures do not include this criterion. [29-31] To the best of our knowledge, no specifically developed or tested metabolome sampling methodology for the Gram-positive model organism *B*. *subtilis* that includes the EC determination has been reported. Even though claims have been made concerning the effectiveness of various sampling methods [5,32], no data concerning modification or development of methods were presented. There is only one report [26] describing an acceptable EC of 0.79 ± 0.04 for *B*. *subtilis* in mid logarithmic growth phase. However, no specific metabolome sampling procedures were tested or described because the samples were collected according to a protocol developed for transcriptome analysis [33]. This does not facilitate the assessment of the procedure for other metabolome investigations.

In the present work, we describe the development of a reproducible methodology to obtain intracellular metabolome data for *B*. *subtilis*. For this purpose, different analytical techniques were applied in order to analyze as many chemically different metabolites as possible. Whereas amino acids, organic acids and small sugar-phosphates were analyzed by GC-MS, nucleosides, nucleotides, sugar-phosphates and co-factors were determined by LC-MS analysis and extracellular metabolites via ^1^H-NMR measurements. Numerous key steps during metabolomic sample preparation and processing were critically tested, including a fast sampling step, extraction of intracellular metabolites, washing of cells, cell disruption, and leakage of metabolites during an attempted liquid nitrogen cooling step before fast vacuum dependent filtration. Because it has been reported that the EC is also influenced by the type of extraction procedure [23], different methods were compared in order to identify the best; whereby not the highest yield of metabolites but rather the highest EC was of primary importance. In addition, we were able to demonstrate that with respect to the EC the culture medium composition also influences the outcome. Finally, our methodology was optimized to achieve an EC within the physiological range and to obtain a maximal amount of extracted metabolites.

## Results

### Cell disruption

**Figure 1:**
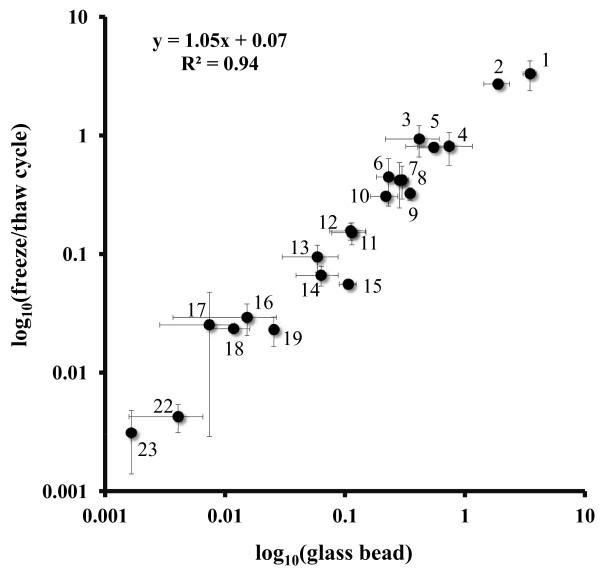
**Metabolite correlation plot; glass bead cell disruption plotted against freeze/thaw cycle cell disruption.** The correlation plot is created of the relative metabolite amounts of (1: ATP, 2: ADP, 3: AMP, 4: Fructose-1,6-bisphosphate, 5: UDP, 6:UTP, 7: GTP, 8: NADP, 9: GDP, 10: IDP, 11: UMP, 12: GMP, 13: IMP, 14: CDP, 15: FAD, 16: CMP, 17: NADPH, 18: NAD, 19: XMP, 20: NADH, 21: CoA, 22: CTP, 23: C6-sugar-phosphate). Relative metabolite amounts are calculates by the integral of the m/z signal of each metabolite related to the m/z internal of the internal standard Br-ATP, gained by glass bead (x-axis) cell disruption compared to freeze/thaw cycle (y-axis) cell disruption. Both axes are log_10_ scaled.

Rapid, efficient, and gentle cell disruption is a key prerequisite for metabolite extraction in metabolome analyses. To optimize the cell disruption by organic solution treatment (60% ethanol) combined with a freeze-thaw cycle by liquid nitrogen, the recoveries of various, chemically diverse intracellular metabolites were compared to those obtained by using the same protocol but with an additional glass bead cell disruption step [23]. The correlation coefficient of 0.94 (Figure 1) demonstrates that both methods do not differ significantly in terms of efficiency. Moreover, the regression coefficient of 1.05 indicates that there is no need for an additional glass bead cell disruption step for *B*. *subtilis* metabolome analyses.

### Extraction of intracellular metabolites – GC-MS analysis

Extraction solutions used in metabolome analyses should be optimized for maximal metabolite extraction efficiency and minimal artificial metabolite modification during sample preparation. To identify the most appropriate extraction solution, GC-MS and LC-MS measurements were carried out. To minimize variation between the biological samples, cell sampling was always performed during the exponential growth phase (OD_600_ = 0.5).

By use of GC-MS, efficiencies of the following extraction solutions were compared: boiling water, boiling 60% (w/v) ethanol, cooled 60% (w/v) ethanol, cooled 60% (w/v) methanol, 1 M formic acid, 100% acetonitrile, and a mixture of methanol, water, and chloroform (4:2:4).

**Figure 2:**
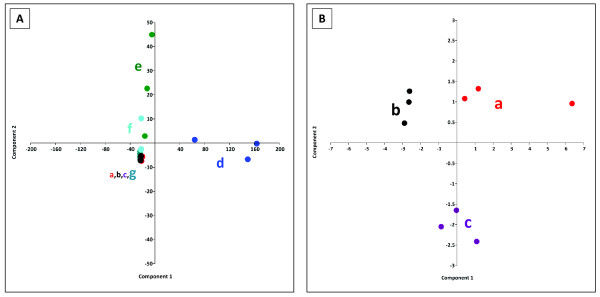
**Principal component analysis (PCA) of the relative metabolite amounts obtained after different extraction methods.** The PCA was created by PAST (Palaeontological Statistics). PAST settings were as follows: Matrix: Var-covar and Boot N: 0 .**A)** PCA of all GC-MS based results of the extraction efficiency tests. a (red): boiling ethanol, b (black): cooled methanol, c) (purple): cooled ethanol, d (dark blue): boiling water, e (green): methanol-chlorofom-water-mixture, f (cyan): acetonitrile, g (light blue): 1M formic acid (Principal component (PC)1: 93.35% variance and PC2: 4.76% variance) **B)** PCA of the GC-MS based result for the extraction power of a) boiling ethanol, b) cooled methanol, c) cooled ethanol (PC1: 63.90% variance and PC2: 18.66% variance).

The principal component analysis (PCA) shown in Figure 2 demonstrates clear differences between the extraction efficiencies of the solutions, whereby the use of boiling ethanol, cooled ethanol and cooled methanol yielded more similar results than the solution containing chloroform. Even though it is obvious that these different hydrophilic extraction solutions yield more similar outcomes compared to a hydrophobic solution, a cluster separation is possible if the PCA calculation is based exclusively on the results of these three hydrophilic extraction methods (Figure 2B).

**Figure 3:**
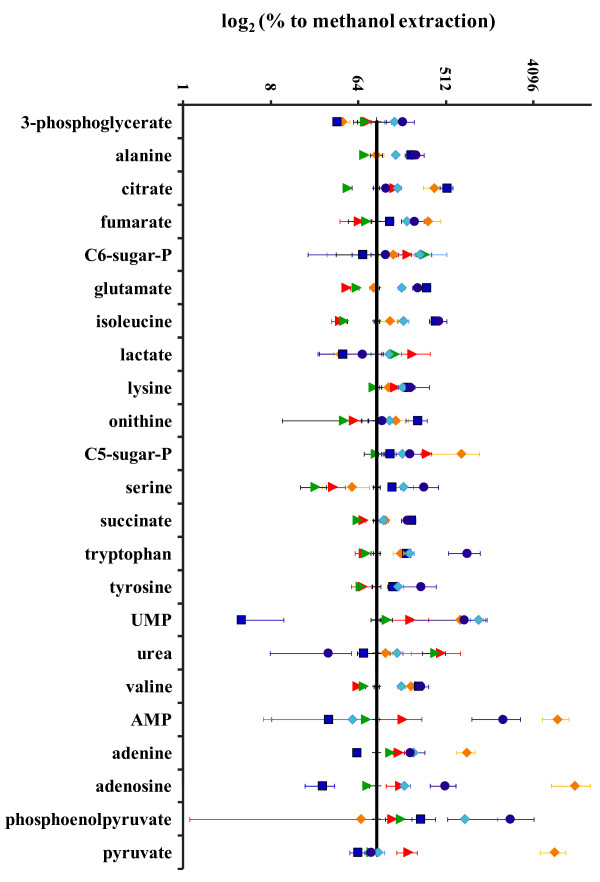
**Comparison of the detected relative metabolite amounts via GC-MS measurement.** Comparison of the detected relative metabolite amounts after several tested extraction techniques related to cooled 60% (w/v) methanol (black line at 100%). Boiling water (yellow), boiling (red) so as cooled (green) 60% (w/v) ethanol, 1 M formic acid (dark blue), 100% acetonitrile (light blue) and methanol/water/chloroform (4:2:4) extraction (purple). Measurements were performed by GC-MS and for comparisons of the extraction potential, 60% (w/v) methanol extractions were carried out in parallel for each tested extraction solution. C5-sugar-P and C6-sugar-P refers to sugar-phosphates containing 5 or 6 carbon atoms respective. The y-axis is log_10_ scaled.

A comparison of the metabolite amounts obtained with the different extraction solutions are presented in more detail in Figure 3.

In the case of 1 M formic acid, high extraction efficiency in particular for citrate, isoleucine and phosphoenolpyruvate (PEP) was achieved. Mixtures of methanol, water and chloroform successfully extracted a large set of metabolites with good efficiency. Boiling water-based extraction yielded high amounts of fumarate, citrate, sugar-phosphate(s) containing 5 carbon atoms, as well as adenine, adenosine and AMP. As described for *Staphylococcus aureus*, this might be caused by the fact that, aside from the sampling and quenching technique, the extraction solution also effects metabolism arrest [23]. Boiling water treatment, in contrast to organic extraction solutions, may only cause reversible inactivation of many enzymes, resulting in more pronounced hydrolysis of ATP due to residual enzyme activity that is reflected in higher amounts of lower-grade phosphorylated compounds. On the other hand, the latter could alternatively be generated by high temperature-induced ATP decomposition during the boiling-water based extraction. To investigate this further, LC-MS measurements were done to detect various nucleotides.

### Centrifugation-based sampling versus fast filtration sampling

In order to find a sampling procedure which introduces the fewest metabolic changes as indicated by the EC, two methods based on either a centrifugation step or a fast vacuum-dependent filtration were compared. The latter was modified by cooling the sample in liquid nitrogen before filtration to arrest metabolism. To determine the energy charge and to evaluate the extraction efficiency of different organic solutions for nucleotides, cofactors and sugar-phosphates, an IP-LC-MS method was used.

*B*. *subtilis* cultures were harvested in exponential growth phase (OD_600_ = 0.5) and metabolites were extracted by using 60% cold ethanol. As the centrifugation method needs at least 5 min even without a further washing step, results obtained with this procedure were subsequently defined as baseline and compared to those obtained by the fast vacuum filtration methods, which take maximally 30 sec without a washing step. As expected, sampling *via* centrifugation resulted in an energy charge of 0.36 ± 0.10, which was clearly lower than the 0.43 ± 0.10 obtained by fast filtration with washing.

**Figure 4:**
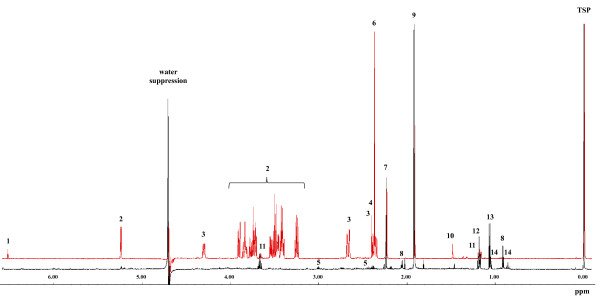
**Extracellular metabolites obtained by **^**1**^**H-NMR measurement (red: exponential growth and black: stationary growth).** 1) fumarate, 2) glucose, 3) malate, 4) succinate, 5) 2-oxoglutarate, 6) pyruvate, 7) unknown, 8) isovaleric acid, 9) acetate, 10) 2-amino-isobutyrci acid, 11) ethanol (could be a contamination), 12) unknown, 13) isobutyric acid, 14) 2-methylbutyric acid. Further small amounts of e.g. butandiol, acetoine, valine and leucine could be detected at some time points during cell growth.

To further optimize the EC by reducing the sampling time, samples were subsequently collected without washing because the washing step during filtration is time consuming for *B*. *subtilis* cells grown in minimal medium. Indeed, omitting the washing step further increased the EC (0.71 ± 0.04). Since the cultivation medium (M9) only contained glucose and malic acid and no additional amino acids, it seemed justifiable to omit the washing step. However, it was also necessary to consider possible contamination of the measured intracellular metabolome by excreted metabolites during analysis and interpretation. Except from small amounts of valine and leucine, no amino acids were detected in the exo-metabolome by GC-MS and ^1^H-NMR analysis, while fumarate, succinate, pyruvate, 2-methylbutyric acid and acetate were clearly secreted (Figure 4). On the other hand, intracellular 2-methylbutyric acid was not detected via GC-MS, neither during exponential growth nor in the stationary phase, indicating that no significant amounts of extracellular metabolites adhere to the cells after filtration.

Although the notable EC increase that was achieved by omitting the washing step, it has been suggested that it is possible to increase the EC even further. For additional optimization of the sampling procedure, the fast vacuum-dependent filtration method was extended by a sample cooling step in liquid nitrogen immediately after cell sampling and before filtration. Indeed, introduction of the additional cooling step in liquid nitrogen resulted in a further increased EC of 0.81 ± 0.03, indicating improved metabolism arrest during filtration (Table 1).

**Table 1:** Overview of the energy charge obtained by the different investigated methods

**Sampling method**	**Energy charge**
**5 min centrifugation, without washing (cooled 60% EtOH)**	0.36 ± 0.076
**Filtration, including washing (cooled H**_**2**_**O)**	0.23 ± 0.140
**Filtration, including washing (cooled MeOH/H**_**2**_**O/CHCl**_**3**_**-mixture)**	0.28 ± 0.129
**Filtration, including washing (boiling 60% EtOH)**	0.19 ± 0.055
**Filtration, including washing (cooled 60% EtOH)**	0.43 ± 0.077
**Filtration, including washing (cooled 60% EtOH ⇨ CHCl**_**3**_**)**	0.41 ± 0.069
**Filtration, including washing (cooled 60% EtOH ⇨ H**_**2**_**O)**	0.43 ± 0.099
**Filtration, without washing (cooled 60% EtOH ⇨ H**_**2**_**O)**	0.71 ± 0.040
**N**_**2 **_**cooling, filtration, without washing (cooled 60% EtOH ⇨ ACN)**	0.80 ± 0.016
**N**_**2 **_**cooling, filtration, without washing (cooled 60% EtOH ⇨ H**_**2**_**O)**	**0.81 ± 0.033**

The used extraction solutions for each method are indicated in the brackets.

**Figure 5:**
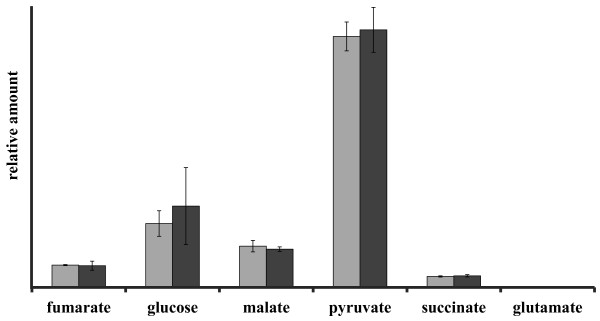
**Cell leakage during liquid nitrogen cooling.** Relative amount (related to the integral of the internal standard ribitol) of extracellular metabolites in the supernatant after direct sterile filtration compared to those in the supernatant after liquid nitrogen cooling. Light grey: direct filtration, and dark grey: after liquid nitrogen cooling.

During this additional cooling step, special care was taken to avoid complete freezing of the samples, since this could cause cell lysis and subsequent leakage of intracellular metabolites. To rule out significant cooling-induced cell lysis, a comparison of metabolites present in the supernatant after fast filtration sampling with or without the additional cooling step was made by GC-MS [34]. No significant differences between the metabolite profiles of both supernatants were found, where in particular the absence of detectable amounts of glutamate in both samples indicated the absence of pronounced cell lysis (Figure 5).

### Extraction of intracellular metabolites – LC-MS analysis

Intracellular metabolites prepared by using cold water, cold 60% ethanol, cold 100% acetonitrile, or a mixture of methanol, water and chloroform (4:2:4) as extraction solutions, respectively, were also analyzed by LC-MS analysis. These solutions yielded the highest metabolite amounts during the GC-MS based extraction solution investigations. In contrast to the GC-MS measurements, the water-based extraction was performed with cold instead of boiling water because the boiling process was suspected of causing metabolite decomposition.

**Figure 6:**
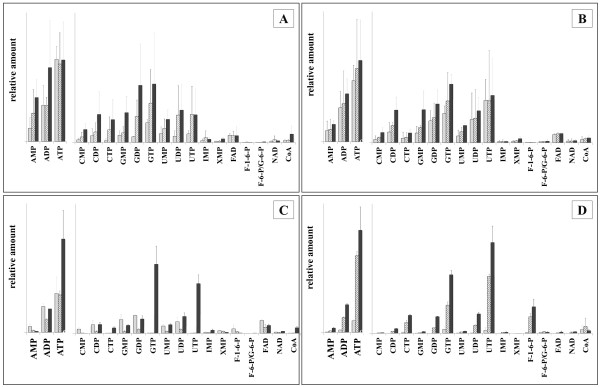
**Comparison of the extraction power of several tested extraction techniques, detected via LC-MS measurement.** Y-axis values are not indicated, since all values are relative abundances related to the integral of the internal standard Br-ATP. F-1,6-P: fructose-1,6-bisphosphate and F-6-P/G-6-P: sum of the relative amount of glucose-6-phosphate and fructose-6-phosphate. **A)** ■ Light gray; cooled 60% ethanol, ▤ striped; mixture of methanol/water/chloroform, ■ dark gray; cooled water. **B)** ■ Light gray; cooled water, ▤ striped; two step extraction by first cooled 60% ethanol and a subsequent 100% chloroform extraction, ■ dark gray; two step extraction by first cooled 60% ethanol and a subsequent 100% water extraction. **C)** ■ Light gray; two step extraction by first cooled 60% ethanol and a subsequent 100% water extraction, ▤ striped; cooled 60% ethanol, ■ dark gray; liquid nitrogen cooling followed by a two step extraction by first cooled 60% ethanol and subsequent a 100% water extraction. **D)** ■ Light gray; 100% cooled acetonitrile, ▤ striped; liquid nitrogen cooling followed by a two step extraction by first cooled 60% ethanol and a subsequent 100% acetonitrile extraction, ■ dark gray; liquid nitrogen cooling followed by a two step extraction by cooled first 60% ethanol and a subsequent 100% water extraction.

Although the cold water extraction resulted in high amounts of nearly all identifiable metabolites, the obtained EC of 0.23 ± 0.14 for this procedure was very low. Similarly, the methanol–water-chloroform-mixture-based extraction resulted in high abundances of the extracted metabolites, but a low EC of 0.28 ± 0.13. The ethanol-based extraction procedure produced a somewhat higher EC of 0.43 ± 0.08 (Table 1). Furthermore, as already observed in the GC-MS measurements, the latter procedure yielded lower extraction efficiencies (Figure 6A).

Putting these results together, we decided to perform an additional extraction step with water or chloroform after that using 60% cooled ethanol since these solutions allowed for the highest metabolite yields during both GC-MS and LC-MS measurements.

The two-step extraction procedure resulted in a higher amount of detectable intracellular metabolites compared to the extraction that used exclusively 60% cold ethanol (Figure 6B).

However, the EC still remained low for all mentioned extraction methods, underpinning the necessity of further improvement. The optimization steps demonstrated that sampling should always be carried out without washing and should use a liquid nitrogen cooling step, allowing for higher EC values (Table 1, Figure 6C). Thus, the two step extraction, first with 60% cold ethanol and second with cold water was performed after cells sampling by the extended filtration method. This resulted in a clear increase in the EC-value to 0.81 ± 0.03. As demonstrated by GC-MS, the extraction with acetonitrile also resulted in high yields of metabolites. Hence, this extraction procedure was now used as second extraction step, combined with the extended filtration method and liquid nitrogen cooling. The observed EC increase again confirmed that introducing a cooling step before filtration obviously causes efficient metabolism arrest (Table 1). However, the obtained amounts of intracellular metabolites were lower compared to extraction with 60% ethanol followed by water extraction (Figure 6D). Thus, the latter extraction method was defined as the most suitable method for *B*. *subtilis* metabolome analyses.

By using this protocol, several intracellular *B*. *subtilis* metabolites were detected, including a number of amino acids and organic acids determined by GC-MS, and nucleotides, cofactors and sugar-phosphates determined by LC-MS. Moreover, analysis of LC-MS data obtained from *B*. *subtilis* in the stationary growth phase (retention time 10–50 min) by online XCMS [9,35] allowed for the detection of more than 750 molecular masses as listed in Additional file 1: Table S1. Of these, about 260 isotopes and 115 adducts were found, while about 30 of these adducts were isotope-adducts. However, only a few of these masses could be identified.

### Intracellular metabolite sampling after cultivation in LB media

The protocol described above was developed for metabolome analysis of *B*. *subtilis* grown in chemically defined medium. However, this procedure is not suitable for *B*. *subtilis* grown in LB or other complex media, where the washing step after fast-filtration based sampling is essential for clear differentiation of intra- and extracellular metabolites and cannot be omitted. In order to validate the usability of the method for cells grown in complex media, we cultivated *B*. *subtilis* in LB medium and performed sampling with and without the washing step. The obtained EC amounted to approximately 0.85 for both procedures and therefore reflected the absence of pronounced metabolic stress. This demonstrated that for cells grown in complex media, the fast-filtration based sampling protocol can be used without omitting the additional washing step.

### Final work flow

**Figure 7:**
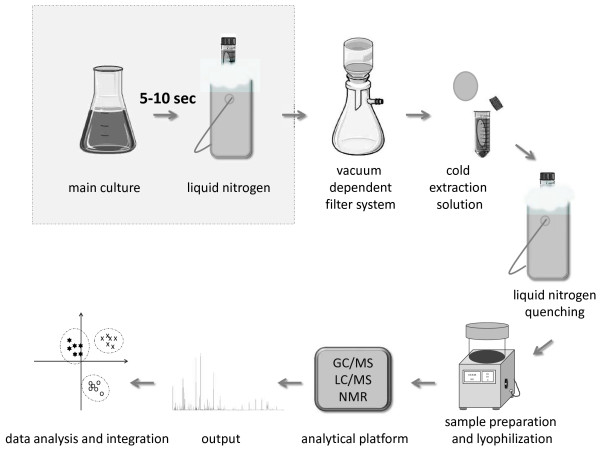
**Workflow.** Workflow for quenching, cell sampling including medium separation, cell disruption and extraction of metabolites by organic solution, lyophilization and analysis of the metabolites occurring from *Bacillus subtilis* cultures.

In this study we optimized single workflow steps in order to produce meaningful metabolome data for *B*. *subtilis*. It became clear that common sampling methods such as cooled centrifugation and fast-vacuum dependent filtration are not applicable for metabolome analysis of *B*. *subtilis* without modifications. Therefore, fast-vacuum dependent filtration sampling was optimized by a preceding sample cooling step by using liquid nitrogen immediately after cell removal from the culture to induce metabolism arrest. After separation of the cells from the medium by fast-vacuum dependent filtration, bacteria-loaded filters were directly transferred to the cold 60% ethanol extraction solution, followed by immediate freezing of the samples in liquid nitrogen. Subsequently, samples were placed on ice and cell disruption and simultaneous metabolite extraction were carried out by vortexing and shaking the sample ten times in succession. A centrifugation step separated the supernatant, which contained the extracted metabolites from cellular debris, and a second, aqueous extraction of the pellet followed. The ethanolic and aqueous supernatants were combined and stored at -80°C. An overview on the optimized protocol is given in Figure 7.

## Discussion

This study confirms the importance of a careful assessment of a metabolome sampling methodology, customized for the specific organism of interest. The need for an optimized, tailor-made individual protocol for each analyzed microbial organism is possibly based on specific differences in the compositions of cell wall and cell membrane as well as different culture conditions. In this work, the influence of specific cultivation conditions was confirmed by the observation of clear differences between the EC obtained after washing the cells grown in minimal or in complex LB media. Aside from the necessity to develop optimized methods for each individual organism under investigation, Schaub et al. (2006) even went so far to suggest that, due to the different metabolite localizations in a cell, it seems unrealistic to use only one protocol for comprehensive metabolome analysis [36]. Even if this might be true, the available analytical techniques do not allow for taking the metabolite location in the cell into account. The newly developed method of MALDI mass spectrometry imaging may make this possible [37]. Nevertheless, the different performances of the various extraction methods could be partially caused by differences in the intracellular metabolite environments such as pH and more hydrophilic vs. hydrophobic milieus. Furthermore, the differencing chemical characteristics of the metabolites might influence the results of the extraction solution analyses. The PCA in Figure 2 clearly illustrates that the outcomes depend on the type of extraction solutions. These differences make it difficult to choose the most useful extraction method. In some cases the main goal is a high yield of intracellular metabolites. This might be acceptable for metabolites with a low turnover rate, such as e.g. amino acids, but for a global metabolite study that also includes short-living metabolites like ATP, the major focus should be a fast sampling technique to avoid stress during sampling. Hence, this study emphasizes the importance of the determination of the EC to evaluate the varying outcomes. Even if a given methodology results in a high amount of intracellular metabolites, this does not guarantee the generation of physiologically meaningful metabolome data with regard to metabolites with a high turnover rate. Confirming this it was demonstrated that besides the sampling and quenching procedure, the extraction solutions also influence the EC. One reason could be reversible or non-reversible enzyme inactivation, depending on the used extraction solution as assumed previously [23,36]. The varying outcomes for the different extraction methods shown in Figure 2, 3 and 6 further indicate that besides the metabolites AMP, ADP and ATP that influence the EC, the amounts of the other metabolites are also dependent on the sampling and extraction procedure. Investigations to study the recovery of metabolites after fast-vacuum filtration followed by ethanolic extraction have been described previously by Meyer and colleagues [23]. Thus, the focus of the final workflow is primarily to obtain physiological meaningful data with an EC in the range described by Atkinson [24]. If latter criterion is guaranteed, the method that results in the highest yield of metabolites was chosen.

Even though this protocol provides the possibility for further optimized metabolome studies on *B*. *subtilis*, further challenges in metabolomics are apparent. The fact that only a fraction of the masses extracted by XCMS can be identified underlines the difficulty of metabolite identification and the need for the developed of new approaches for precise metabolite identification. Even by high resolution mass spectrometry, unambiguous metabolite identification is not always possible because some metabolites have exactly the same mass (e.g. glucose-6-phosphate and fructose-6-phosphate or succinyl-CoA and methyl-malonyl-CoA). Although large online databases such as the human metabolome database [38], the yeast metabolome database [39] and METLIN [40] exist, mostly the measurement of pure standard compounds is necessary for targeted metabolomics. In this context, the main problem is the limited commercial availability of some metabolites [32].

Hence, besides well-developed sampling protocols and established analytical methods the current challenge for targeted metabolomics is the identification of unknown metabolites.

## Conclusion

This study describes the development of an optimized sampling methodology for *B*. *subtilis* metabolome analyses. To the best of our knowledge, this is the first *B*. *subtilis* metabolome analysis protocol that takes into account the EC. Except for work described by Coulier el al. (2006) [26], the EC for *B*. *subtilis* has not be described in the literature [32] or is below the range described by Atkinson [24]. The different outcomes seen with different extraction solutions as well as the varying results depending on the sampling protocol indicates that not only is the EC influenced by the chosen metabolome sampling protocol, but the whole metabolite profile as well. This underlines the importance of specifically developed protocols.

Finally, the described approach offers the possibility of a global view of the intracellular metabolome of *B*. *subtilis*. This would enable, for example, screening of metabolically interesting mutants or analyzing the dependency of the intracellular metabolome on growth conditions or environmental stresses.

## Methods

### Cultivation

A Difco sporulation medium (DSM) plate of *B*. *subtilis* 168trp^+^[41] was prepared from frozen stocks (-80°C, in 15% (v/v) glycerol) and incubated at 37°C for 24 h. From latter plate LB plates were prepared and incubated at 37°C for 24 h. For the pre-culture, 5 ml LB medium including 1 μg/ml erythromycin and 5 μg/ml chloramphenicol antibiotics were inoculated with a colony from the abovementioned LB plate. The cells were incubated for 4 h at 37°C and 240 rpm. The LB cultures were used to inoculate five different diluted (1:25000, 1:30000, 1: 35000, 1:40000 and 1:50000) overnight culture in chemical defined M9 medium containing 0.1% glucose and 0.1% malic acid. The overnight cultures were incubated for 14 h at 37°C and 300 rpm. Before inoculation of the main culture to an OD_600nm_ of 0.05, the overnight culture was tested to ensure it was in exponential growth phase (OD_600nm_ = 0.4-0.8). The main culture was incubated in M9 medium containing 0.1% glucose and 0.1% malic acid in a shake flask under aerobic conditions at 37°C and 300 rpm.

Cultivations in LB (lysogenic broth) medium were carried out as described above for M9 medium cultivation, whereas overnight culture and the main culture were incubated in LB medium.

### Intracellular metabolite sampling

Additional file 2: Table S2 (additional information) presents a list of all investigated sampling, extraction and cell disruption methods.

### Centrifugation sampling

20 ml of the main culture was harvested in exponential growth phase (OD_600_ = 0.5) and centrifuged for 5 min at 13000 rpm and 0°C. Afterwards the supernatant was discarded and 5 ml of cold 60% ethanol (w/v) including 100 nmol brom-adenosin-5’-triphosphat (Br-ATP) as internal standard was added to the cell pellet.

### Filtration sampling

20 ml of the main culture was harvested in exponential growth phase (OD_600_ = 0.5) via fast vacuum-dependent filtration, modified after [23]. The upper part of the filtration system was cooled at -20°C for 10 min before sampling. The concentration of the isotonic washing solution was changed to 0.8% NaCl for adaption to the growth conditions for *B*. *subtilis* grown in M9 medium (for all samples in which the washing step was carried out). After LB cultivations a 0.9% NaCl solution was used for the washing step. Furthermore a liquid nitrogen pre-cooling step was appended to selected samples in order to increase the energy charge. For this purpose 20 ml of the main culture was poured into a 50 ml plastic centrifuge tube and cooled with liquid nitrogen for 10 sec to stop enzymatic activity and prevent the turnover of metabolites (sample temperature after cooling 9±2°C). The plastic centrifuge tube was dipped periodically (ten times and max. 10 sec in total) in and out of the liquid nitrogen by stages and shaken carefully in between in order to avoid freezing of the sample and consequent metabolite leakage caused by cell lysis. Subsequently the cooled culture was filtered and following the filter including the cells was added to the pre-cooled (30 min at -20°C) extraction solution. Immediately after, metabolism was quenched by liquid nitrogen. The frozen samples were stored at -80°C until further treatment.

### Extracellular metabolite sampling

2 ml cell culture was sterile filtered (ø pore 0.45 μm, Filtropur S^®^, Sarstedt) rapidly into a 2 ml tube and stored at -20°C as described previously [42].

### Metabolite extraction

In this work, eleven methods for metabolite extraction were investigated as listed in Table 2.

**Table 2:** All investigated extraction solution and the downstream analytic used are noted for each extraction method

**Extraction solution**	**Applied method**
**Cooled methanol, water and chloroform mixture (4:2:4)**	GC-MS and LC-MS
**Cooled water, 100%**	LC-MS
**Boiling water, 100%: the filter including cells was added to a plastic**	GC-MS
**Centrifuge tube and the sample were heated for 10 min in a 100°C water bath.**	
**Cooled 60% (w/v) methanol**	GC-MS
**Cooled 60% (w/v) ethanol**	GC-MS and LC-MS
**Boiling 60% (w/v) ethanol, accomplished as for boiling water.**	GC-MS and LC-MS
**Cooled 1 M formic acid**	GC-MS
**Cooled 100% acetonitrile**	GC-MS and LC-MS
**Cooled ethanol 60% (w/v) followed by a second water extraction**	LC-MS
**Cooled ethanol 60% (w/v) followed by a second chloroform extraction**	LC-MS
**Cooled ethanol 60% (w/v) followed by a second acetonitrile extraction**	LC-MS

GC-MS analyses were preformed for extraction solution efficiency investigations with regard to amino acids, organic acids, small sugar phosphates and nucleoside monophosphate. IP-LC-MS analyses were mainly preformed to evaluate the energy charge of samples depending on the applied sampling and extraction method and to investigate the quantity of the cell disruption method. Moreover the IP-LC-MS data were used to evaluate the extraction efficiency of nucleotides, fructose-1,6-bis-phosphate, frucose-6-phosphate/glucose-6-phosphate, FAD, NAD and CoA.

For each extraction method, 5 ml of the extraction solution including the internal standard (100 nmol Br-ATP for LC-MS and 20 nmol ribitol and norvaline for GC-MS analysis) was added to a 50 ml plastic centrifuge tube. The cold extraction solutions were pre-cooled at -20°C for 30 min (water was pre-cooled at 4°C for 30 min). During the sampling procedure, the solutions were kept on ice (≤ 6°C). Each experiment was performed in triplicate. For comparison of the extraction methods, all samples were taken at an OD_600nm_ of 0.5 and the methanol extraction was carried out in parallel for all GC-MS experiments.

### Cell disruption - glass bead method

The glass bead cell disruption method was carried out as described by Meyer et al. [23]. Samples were thawed on ice (≤ 6°C) and splitted to 5 x 1 ml. Each 1 ml extraction solution containing the cell sample was added to screw cap micro tube (SARSTEDT) filled with 0.5 ml glass beads (Sartorius AG, diameter 0.10–0.11 mm).

Next the cells were disrupted in a homogenizer (Precellys 24) using 2 cycles for 3 s at 6800 rpm. The glass beads and the cell debris were separated from the supernatant by centrifugation for 5 min at 4°C and 13000 rpm, the aliquots were combined, and the glass beads were washed once with double-distilled water. The washing solutions were added to the metabolite extracts. The metabolite containing samples were restocked with double-distilled water to a final organic solution concentration of 10% and stored at -80°C prior to lyophilization.

### Cell disruption – freeze/thaw cycle

Cells disruption was carried out by the extraction solution and a freeze/thaw cycle after filtration. For this method, the N_2_ frozen and at -80°C stored samples were thawed on ice (≤ 6°C), vortexed and shaken 10 times alternately and centrifuged for 5 min at 4°C and 13000 rpm. In case of the two step extraction methods, the supernatant were filled to a new 50 ml plastic centrifuge tube. The pellet was once more extracted by the second extraction solution (water, chloroform or acetonitrile), vortexed and shaken 10 times alternately and centrifuged for 5 min at 4°C and 13000 rpm. The supernatants were combined and the pellet was discarded.

After metabolite extraction and cell disruption, the supernatant including the extracted metabolites were restocked with double-distilled water to a final organic solution concentration of 10% and stored at -80°C prior to lyophilization. All steps were carried out on ice (≤ 6°C).

### Analytical methods

#### NMR

400 μL of the sterile filtered (ø pore 0.45 μm, Filtropur S^®^, Sarstedt) extracellular metabolite sample was buffered to pH 7.0 by addition of 200 μL of a sodium hydrogen phosphate buffer (0.2 mM [pH 7.0], including 1 mM TSP)) made up with 50% D_2_O to provide a nuclear magnetic resonance (NMR)-lock signal. The metabolite containing samples were measured by a 600.27 MHz NMR at 310 K using a Bruker AVANCE-II 600 NMR spectrometer (Bruker Biospin GmbH, Rheinstetten, Germany). A modified 1D-NOESY pulse sequence was adopted and a total of 64 free induction decays (FID scans) were collected using a spectral width of 30 ppm for a one-dimensional spectrum. AMIX 3.9 was used for data processing and analysis.

Intracellular metabolites were measured by GC-MS and LC-MS. Prior to analysis samples were lyophilized at 0.54 mbar and -54°C (Christ Alpha 1–4).

#### GC-MS (ES, quadrupol)

GC-MS analysis was performed for amino acids, organic acids, fatty acids, sugars, nucleobases, intermediates from the glycolysis and some nucleoside monophosphates. Completely lyophilized samples were derivatized for 90 min at 37°C with O-methylhydroxylamin-hydrochlorid (MeOX) and 30 min at 37°C with N-methyl-N-(trimethylsilyl)-trifluoroacetamid (MSTFA) and centrifuged for 2 min at room temperature. The supernatants were transferred into glass-vials prior to GC-MS analyses as described previously [43]. Data analysis was performed by MetaQuant [44]. For determination of the relative metabolite amounts, the mass spectrometric base peak of each metabolite was integrated and normalized to the base peak integral of the internal standard ribitol.

#### IP-LC-MS (ESI-TOF)

The detection of nucleotides, nucleosides, sugar-phosphates and cofactors was performed by an ion-pairing-LC-MS (IP-LC-MS) method as described earlier [45] (Agilent HPLC System 1100; Agilent Technologies, USA). Completely dried samples were dissolved in 100 μl double-distilled water and centrifuged for 2 min (13.000 rpm, 2°C). The supernatants were transferred into glass vials with micro inserts for small volume injections.

Chromatographic separation was performed using a RP-C_18_ Waters^®^ Symmetry-Shield column (150x4.6 mm, 3.5 μm) with a C_18_ waters^®^ precolumn. The gradient flow rate was 0.3 ml/min. The solutions were as follows:

A) 5% methanol and 95% water, containing 10 mM tributylamine as the ion-pairing (IP) reagent and 15 mM acetic acid. NH_3_ were used for pH adjustment to pH 4.9.

B) 100% methanol.

The HPLC gradient was as described in [45].

The HPLC was coupled to a micrOTOF mass spectrometer (Bruker Daltonics, Bremen, Germany) operating in ESI negative ionization mode using a mass range from 100 to 2000 m/z.

Data analysis was carried out by QuantAnalysis^®^ (Bruker Daltonics, Bremen, Germany). Relative metabolite amounts were calculated by the m/z integral of each metabolite normalized to the m/z integral of the internal standard Br-ATP.

#### Energy charge determination

For energy charge determination AMP, ADP and ATP have been quantified. The peak areas of the exact m/z of latter metabolites were integrated by QuantAnalysis^®^ (Bruker Daltonik, Bremen, Germany). The peak areas of each extracted ion (m/z) were normalized to the integral of the m/z area of the internal standard Br-ATP. For determination of the calibration equation, different concentrations of pure standards were measured and analyzed in the same manner. Linear regression equations were determined in excel^®^. The EC was calculated by the formula:

$$\frac{\left[\text{ATP}\right]+\frac{1}{2}\left[\text{ADP}\right]}{\left[\text{ATP}\right]+\left[\text{ADP}\right]+\left[\text{AMP}\right]}$$

## Competing interest

The authors declare that they have no competing interests.

## Authors’ contributions

HM elaborated experiments, performed cultivations, all sampling optimization steps, carried out NMR, GC-MS and LC-MS analysis, implemented data analyses and wrote the manuscript. HW performed and optimized cultivation conditions to enable further experiments. ML supervised the experiments and participated in the manuscript draft. All authors read and approved the final manuscript.

## Supplementary Material

Additional file 1: Table S1 Mass list obtained by online XCMS. Click here for file

Additional file 2: Table S2 List of all investigated sampling, cell disruption, leakage and extraction methods. Click here for file
