# Supplementary material for: Methodological approaches to help unravel the intracellular metabolome of Bacillus subtilis
Source: Microb Cell Fact. 2013 Jul 11;12:69. doi: 10.1186/1475-2859-12-69 (PMC3722095; doi:10.1186/1475-2859-12-69)
Supplement: Additional file 2: Table S2 — List of all investigated sampling, cell disruption, leakage and extraction methods. [file 1475-2859-12-69-S2.pdf]

investigation	sampling protocol	cell disruption	extraction solution	analytical method
**extraction solution efficiency**	cold centrifugation	N_2+solvent	cooled methanol, 60% (w/v)	GC-MS
**extraction solution efficiency**	cold centrifugation	N_2+solvent	cooled ethanol, 60% (w/v)	GC-MS
**extraction solution efficiency**	cold centrifugation	N_2+solvent	boling water	GC-MS
**extraction solution efficiency**	cold centrifugation	N_2+solvent	boiling ethanol, 60% (w/v)	GC-MS
**extraction solution efficiency**	cold centrifugation	N_2+solvent	cooled formic acid, 1 M	GC-MS
**extraction solution efficiency**	cold centrifugation	N_2+solvent	cooled acetonitrile, 100%	GC-MS
**extraction solution efficiency**	cold centrifugation	N_2+solvent	cooled methanol, chloroform, water-mixture (4:4:2)	GC-MS
**cell disruption**	cold filtration	N_2+solvent	cooled ethanol, 60% (w/v)	LC-MS
**cell disruption**	cold filtration	homogenizer	cooled ethanol, 60% (w/v)	LC-MS
**sampling and extraction method (EC determination)**	5 min cold centrifugation without washing	N_2+solvent	cooled ethanol, 60% (w/v)	LC-MS
**sampling and extraction method (EC determination)**	cold filtration including washing	N_2+solvent	cooled ethanol, 60% (w/v)	LC-MS
**sampling and extraction method (EC determination)**	cold filtration including washing	N_2+solvent	cooled water	LC-MS
**sampling and extraction method (EC determination)**	cold filtration including washing	N_2+solvent	cooled methanol, chloroform, water-mixture (4:4:2)	LC-MS
**sampling and extraction method (EC determination)**	cold filtration including washing	N_2+solvent	boling ethanol, 60% (w/v)	LC-MS
**sampling and extraction method (EC determination)**	cold filtration including washing	N_2+solvent	cooled ethanol, 60% (w/v) followed by a second water extraction	LC-MS
**sampling and extraction method (EC determination)**	cold filtration including washing	N_2+solvent	cooled ethanol, 60% (w/v) followed by a second chloroform extraction	LC-MS
**sampling and extraction method**	cold filtration including washing	N_2+solvent	cooled ethanol, 60% (w/v) followed by a second water	LC-MS

**(EC determination)**			extraction	
**sampling and extraction method (EC determination)**	cold filtration without washing	N_2+solvent	cooled ethanol, 60% (w/v)	LC-MS
**sampling and extraction method (EC determination)**	N_2 cooling followed by cold filtration without washing	N_2+solvent	cooled ethanol, 60% (w/v) by a second acetonitrile extraction	LC-MS
**sampling and extraction method (EC determination)**	N_2 cooling followed by cold filtration without washing	N_2+solvent	cooled ethanol, 60% (w/v) followed by a second water extraction	LC-MS
**leakage during N_2 cooling**	N_2 cooling followed by cold filtration without washing	N_2+solvent	measurement of the supernatant	GC-MS
**leakage during N_2 cooling**	direct steril filtration	N_2+solvent	measurement of the supernatant	GC-MS
